# Model Selection for Body Temperature Signal Classification Using Both Amplitude and Ordinality-Based Entropy Measures

**DOI:** 10.3390/e20110853

**Published:** 2018-11-06

**Authors:** David Cuesta-Frau, Pau Miró-Martínez, Sandra Oltra-Crespo, Jorge Jordán-Núñez, Borja Vargas, Paula González, Manuel Varela-Entrecanales

**Affiliations:** 1 Technological Institute of Informatics, Universitat Politècnica de València, 03801 Alcoi Campus, Spain; 2 Department of Statistics, Universitat Politècnica de València, 03801 Alcoi Campus, Spain; 3 Internal Medicine Department, Teaching Hospital of Móstoles, 28935 Madrid, Spain

**Keywords:** permutation entropy, sample entropy, approximate entropy, logistic regression, body temperature, signal classification

## Abstract

Many entropy-related methods for signal classification have been proposed and exploited successfully in the last several decades. However, it is sometimes difficult to find the optimal measure and the optimal parameter configuration for a specific purpose or context. Suboptimal settings may therefore produce subpar results and not even reach the desired level of significance. In order to increase the signal classification accuracy in these suboptimal situations, this paper proposes statistical models created with uncorrelated measures that exploit the possible synergies between them. The methods employed are permutation entropy (PE), approximate entropy (ApEn), and sample entropy (SampEn). Since PE is based on subpattern ordinal differences, whereas ApEn and SampEn are based on subpattern amplitude differences, we hypothesized that a combination of PE with another method would enhance the individual performance of any of them. The dataset was composed of body temperature records, for which we did not obtain a classification accuracy above 80% with a single measure, in this study or even in previous studies. The results confirmed that the classification accuracy rose up to 90% when combining PE and ApEn with a logistic model.

## 1. Introduction

A great diversity of time series has been successfully analysed in the last several decades since the widespread availability of digital computers and the development of efficient data acquisition and processing methods: biology time series [1], econometrics records [2], environmental sciences data [3], industrial processes and manufacturing information [4], and many more. The case of non-linear methods, capable of extracting elusive features from any type of time series, is especially remarkable. However, these methods can sometimes be difficult to customize for a specific purpose, and some signal classification problems remain unsolved or scarcely studied. In this regard, this paper addresses the problem of physiological temperature record classification. This problem has only recently begun to be studied [5], with so far only marginally significant differences [6]. Instead of trying to find a single better non-linear optimal measure or parameter configuration, we propose a new approach, based on a combination of several sub-optimal methods.

Electroencephalographic (EEG) and heart rate variability (HRV) records are probably the two types of physiological time series most analyzed in signal classification studies using non-linear methods. The rationale of this scientific popularity is twofold. On the one hand, these records are frequently used in clinical practice, convenient and affordable monitoring devices abound, and a growing body of publicly available data has been created in the last several decades. On the other hand, recently developed non-linear measures suit well the features and requirements of these records with regard to a number of samples and noise levels.

As a consequence, there are a myriad of scientific papers describing successful classification approaches for different types of EEG or HRV signals. For instance, in [7], EEG signals were classified using several entropy statistics under noisy conditions. Despite high noise levels, most of the entropy methods were able to find differences among signals acquired from patients with disparate clinical backgrounds. The study in [8] used two of these measures to group epileptic recordings of very short length, only 868 samples. The work [9] also used EEG records to detect Alzheimer’s disease based on changes in the regularity of the signals. One out of the two methods assessed was able to find significant differences between pathological and healthy subjects. Regarding RR time series, studies such as [10] classified congestive heart failure and normal sinus rhythm again using two entropy measures and assessed the influence of the input parameters on these measures. One of the very first applications of sample entropy (SampEn) was the analysis of neonatal HRV [11]. Other approaches based on ordinal patterns instead of amplitude differences have also been successful in classifying HRV records, in this case for the diagnosis of cardiovascular autonomic neuropathy [12]. In summary, EEG and HRV records have been extensively processed using approximate entropy (ApEn), SampEn, distribution entropy (DistEn), fuzzy entropy (FuzzyEn), permutation entropy (PE), and many more, in isolation or in comparative studies.

Conversely, other biomedical records, such as blood or interstitial glucose, arterial blood pressure, or body temperature data, have not been studied as extensively. Despite their convenience and demonstrated diagnosis potential [13,14], the use of entropy, complexity, or regularity measures is still lacking in these contexts. These records are more often found in clinical settings as single readings instead of time series, and if continuous readings are available, they are usually very short and sampled at very low frequencies. As a result of this lack of scientific literature and data on which further studies can be rooted, the selection of methods and parameters is more difficult. Thus, the quest for suitable classification methods may become a brute force search, and often the results achieved are at the borderline of significance, at most. The question that arises in these cases can be: Are there no method differences because the records from several classes do not exhibit any particular feature or because the method has not been used to its full potential?

Obviously, when a single feature is not sufficient for a clear classification of objects, more features can be included in the classification function, following the general pattern recognition principles for feature extraction and selection. For instance, in [14], in addition to a non-linear measure for early fever peak detection, the classification function used other parameters such as the temperature gradient between core and peripheral body temperature. To detect atrial fibrillation in very short RR records, in [15], the authors proposed adding the heart rate as a predictor variable along with the entropy estimate. In these and other examples, a single non-linear method was combined with other parameters to improve the accuracy of the classifier employed. Along these lines, in this paper, we propose the use of two variables for classifying body temperature records. However, instead of using a single non-linear method combined with other unrelated parameters [15], the main novelty of our work is the utilization of two uncorrelated entropy measures as explanatory variables of the same equation: PE and ApEn/SampEn. There are other works with comparative analyses using several entropy measures independently, and some authors have even recommended applying more than one method together to reveal different features of the underlying dynamics [16], but our method combines more than one measure together in a single function after a correlation analysis. There are a few studies using pattern recognition techniques and more than one entropy statistic, such as [17], to improve the classification accuracy of a single method.

For many years, temperature recordings in standard clinical practice have been limited to scarce measurements (once per day or once per shift), which provides very little information about the processes underlying body temperature regulation [18,19]. For these reasons, physicians are only capable of distinguishing between febrile patients and afebrile patients. However, information from continuous body temperature recordings may be helpful in improving our understanding of body temperature disorders in patients with fever [5,20,21].

ApEn and SampEn are arguably the two families of statistics most extensively used in the non-linear biosignal processing realm, with ApEn accounting for more than 1100 citations in PubMed and SampEn for almost 800. PE is not that common yet, since it is a more recent method, but it is probably the best representative of the tools based on sample order differences instead of on sample amplitude differences, as is the case for ApEn and SampEn. Different values of SampEn/ApEn and PE between healthy individuals and patients with fever are likely to reflect subtle changes in body temperature regulation that may be more relevant than the mere identification of a fever peak. It seems reasonable to believe that the process of body temperature regulation may be altered during infectious diseases and it may return to normal during the recovery phase [22]. Therefore, information obtained by non-linear methods could be useful to evaluate the response to antimicrobial treatments or to adjust the length of those treatments.

Each method separately provides a borderline temperature body time series classification, as is the case in many other studies, but the two combined improve its accuracy significantly. The results of our study show that logistic models including SampEn/ApEn and PE have and accuracy that is acceptable for classifying temperature time series from patients with fever and healthy individuals. The ability of the models developed in this work to classify body temperature time series seems to be the first step in giving temperature recording a more significant role in clinical practice. As has been proved with other clinical signals like heart rate or glycaemia [10,13], many diseases reflect a deep disturbance of complex physiologic systems, which can be measured by non-linear statistics. This scheme could therefore be exported to other similar situations where several methods are assessed but none of them reaches the significance level desired. The solution to most of these problems probably lies in a similar approach to that described in the present paper, whose main contributions are an improvement in body temperature classification accuracy and the introduction of a logistic model to perform such a classification.

## 2. Materials and Methods

### 2.1. Entropy Measures

The input to all the entropy measures used in this study is a normalized time series of length *L*, $x=\left\{x_{1},x_{2},x_{3},\ldots ,x_{L}\right\}$, from which embedded sequences of length *m* starting at sample *t* can be extracted as $x_{t}=\left\{x_{t},x_{t+1},x_{t+2},\ldots ,x_{t+\left(m-1\right)}\right\}$, with 1≤t≤L−(m−1). With this input, the main steps to compute ApEn, SampEn, and PE are defined next. The references included can be consulted for further details.

#### 2.1.1. Approximate Entropy

ApEn is a very successful entropy measure for signal classification that was first introduced in [23]. Given the input time series x, a distance *d* between two embedded sequences $x_{i}=\left\{x_{i},x_{i+1},x_{i+2},\ldots ,x_{i+\left(m-1\right)}\right\}$ and $x_{j}=\left\{x_{j},x_{j+1},x_{j+2},\ldots ,x_{j+\left(m-1\right)}\right\}$ is defined as $d_{ij}=\max(|x_{i+k}-x_{j+k}\left|\right),0\leq k\leq m-1$. For each pair 1≤i,j≤L−m+1, this distance has to be computed. A variable termed $C_{ij}$, is assigned 1 each time the distance $d_{ij}$ between the associated two sequences is lower than a predefined threshold *r*, 0 otherwise:

$$C_{ij}=\left\{\begin{array}{cc} 0 & \mathrm{if}\;d_{ij}\geq r\\ 1 & \mathrm{if}\;d_{ij}<r \end{array}.\right.$$

All the $C_{ij}$ values are averaged to obtain the following statistic:

(1) $$C_{i}^{m}\left(r\right)=\frac{1}{L-m+1}\sum_{j=1}^{L-m+1}C_{ij}.$$

This variable is then log-averaged:

(2) $$\Phi^{m}\left(r\right)=\frac{1}{L-m+1}\sum_{i=1}^{L-m+1}\log\left(C_{i}^{m}\left(r\right)\right),$$

and the process is repeated for m⟶m+1. Finally, ApEn can be obtained as

(3) $$\mathrm{ApEn}\left(m,r,L\right)=\Phi^{m}\left(r\right)-\Phi^{m+1}\left(r\right).$$

#### 2.1.2. Sample Entropy

SampEn was introduced in [24], as an improvement for ApEn. SampEn and ApEn algorithms are quite similar, especially the first steps. The main differences are that self-matches are not computed (j≠i) in Equation (1), since that case is not included in $C_{ij}$:

(4) $$C_{i}^{m}\left(r\right)=\frac{1}{L-m}\sum_{j=1,j\neq i}^{L-m+1}C_{ij},$$

and that variable is now linearly averaged instead (compared to Equation (2)):

(5) $$\Phi^{m}\left(r\right)=\frac{1}{L-m+1}\sum_{i=1}^{L-m+1}C_{i}^{m}\left(r\right).$$

The process is again repeated for m⟶m+1. Finally, SampEn can be obtained as

(6) $$\mathrm{SampEn}\left(m,r,L\right)=-\log\left[\frac{\Phi^{m+1}\left(r\right)}{\Phi^{m}\left(r\right)}\right].$$

#### 2.1.3. Permutation Entropy

Contrary to ApEn and SampEn, PE is based on $x_{t}$ ordinal differences instead of amplitude differences [25]. The subsequences under comparison are not the amplitude of the samples but are the resulting sample indices after repositioning them in ascending order. Initially, before the ordering process takes place, the default index set for $x_{t}=\left\{x_{t},x_{t+1},x_{t+2},\ldots ,x_{t+\left(m-1\right)}\right\}$ is $\left\{0,1,\cdots ,\left(m-1\right)\right\}$. Defining the vector $\pi_{t}$ as the permutation indices of $x_{t}$ once it is re-assembled in sample ascending order, $\pi_{t}=\left\{\pi_{t},\pi_{t+1},\pi_{t+2},\ldots ,\pi_{t+\left(m-1\right)}\right\}$, such that $x_{\pi_{t}}<x_{\pi_{t+1}}<\ldots <x_{\pi_{t+(m-1))}}$, with $\pi_{i}\in \left[0,m-1\right],$ and $\pi_{i}\neq \pi_{j},\forall i\neq j.$ The probability $p\left(\pi_{t}\right)$ of each ordinal pattern can be estimated as its relative frequency, taking into account all the possible m! permutations of *m* symbols (indices), and all the L−m+1 embedded sequences $x_{t}$ of length *m*:

(7) $$p\left(\pi_{t}\right)=\frac{\mathrm{card}(\pi_{t})}{L-m+1}$$

where card() accounts for the cardinality of π, namely, the number of times that order is found in the subsequences. There are potentially up to m! different $\pi_{t}$ patterns, although it is quite usual that some of them have a cardinality of 0. PE can then be computed as the Shannon entropy of the resulting probability distribution:

(8) $$\mathrm{PE}\left(m,L\right)=-\sum_{\forall p\left(\pi_{t}\right)\neq 0}\left(p\left(\pi_{t}\right)\log_{2}p\left(\pi_{t}\right)\right).$$

As for many other entropy statistics, a time scale could be applied to PE. This embedded delay, usually termed τ, is often introduced as a subsampling factor in the input time series [26]. This study only uses the original time scale of the data for all the entropy methods, including PE, and therefore τ=1.

### 2.2. Classification Analysis

The classification is based on a quantitative model using PE, ApEn, SampEn, or a combination of two of them as input variables. Specifically, we have used a logistic regression probabilistic model [27]. The objective is to model the expected two classes of the temperature records (sick/healthy), one coded as 0 and the other coded as 1. One of the strengths of this model is that it does not require the data assumptions of other models, such as normality, linearity, or homoscedasticity, making it less restrictive than other methods such as discriminant analysis [28]. Moreover, this model is almost 10 times less data hungry than other classification techniques such as support vector machines or neural networks. With only 18–23 samples, logistic models are able to achieve a difference between the apparent AUC and the validated AUC smaller than 0.01 [29]. It is also one of the most stable classifiers, yielding consistent results for both training and validation experiments, even with imbalanced datasets [30].

Logistic models have been successfully applied in many time series classification tasks: EEG [31,32], HRV [33,34], as stated in the Introduction section, and others beyond the medical framework such as [35,36,37]. The general expression for this model is

$$p\left(z\right)=\frac{\exp\left(b_{0}+\sum_{i=1}^{q}b_{i}z_{i}\right)}{1+\exp\left(b_{0}+\sum_{i=1}^{q}b_{i}z_{i}\right)}$$

where p(z) is the probability of the predicted class being 1. The variables $b_{i}$ are the regression coefficients, and $z_{i}$ are the quantitative variables, in this case the entropy statistic employed (therefore, q=1 or q=2 if one or two statistics are included in the model, respectively).

Specifically, the model for each case becomes

$$p\left(z\right)=\frac{\exp\left(b_{0}+b_{1}z_{1}\right)}{1+\exp\left(b_{0}+b_{1}z_{1}\right)}$$

for the individual models (univariate model), with $z_{1}$ accounting for the entropy measurement employed, that is, $z_{1}=\mathrm{ApEn}\left(m,r,L\right)$ for the model using ApEn only, $z_{1}=\mathrm{SampEn}\left(m,r,L\right)$ for the SampEn based model, and $z_{1}=\mathrm{PE}\left(m,r,L\right)$ for PE. The parameters $b_{0}$ and $b_{1}$ are the unknowns that the model computes. For the model using two measures (bivariate model), the general expression is

$$p\left(z\right)=\frac{\exp\left(b_{0}+b_{1}z_{1}+b_{2}z_{2}\right)}{1+\exp\left(b_{0}+b_{1}z_{1}+b_{2}z_{2}\right)}$$

with $z_{1}$ and $z_{2}$ accounting for the two measures employed, PE, and either ApEn or SampEn. In this case, there are three parameters to compute: $b_{0},b_{1},$ and $b_{2}$.

The computation of the model was carried out using the R statistical package [38]. The output includes the coefficients stated above ($b_{0},b_{1},b_{2}$), the standard error, the Wald statistic [39], the degrees of freedom, the significance, and the exponentiation of the $b_{0}$ coefficient, which is the odds ratio. These results will be shown in Section 3.

The Akaike information criterion (AIC) [40] is the metric used for model assessment. This statistic is computed for all the models to be compared. The best model is that with the minimum AIC of all the models under comparison. In practical terms, the goodness of fit of the model will be assessed computing the probability of class membership for each record in the experimental database. The optimal model will be that with the minimum number of classification errors or with the maximum classification accuracy. This accuracy will be quantified in terms of specificity and sensitivity. These values can be directly obtained from the ROC curve, changing the threshold point. However, the model obtained using all the time series in the data set may not give a reliable idea of the classification capability of the model (over-fitting risk). It is better to apply the model to a subset not involved in the process of estimating it. Since the dataset is relatively small, we used the leave-one-out (LOO) method [41] and the same statistical package [42], in which all the time series except one for each class are used to estimate the model parameters. Specifically, one time series of each class is held out and not included in the model calculations (test set), and the remaining ones are used to obtain the model (training set). The resulting model is then applied to the unused pair of series in order to evaluate its performance on unseen data. This process is repeated for all the records in the dataset. The overall prediction error is finally obtained by averaging errors from each individual model obtained [43].

The proportion of variance explained by the model is quantified by the Nagelkerke [44] and the Cox–Snell $R^{2}$ [45] coefficients. A value for the Nagelkerke $R^{2}$ greater than 0.5 indicates that the variance is well explained. The minimization criterion is based on the −2 log-likelihood (−2LL) [46], the smallest possible deviance or residual variance. These values will also be reported in the Results section.

### 2.3. Experimental Dataset

The experimental dataset is composed of 30 body temperature records obtained from two groups of individuals. The first group included 16 healthy individuals (10 women and 6 men). They were asked to refrain from taking a shower and to avoid strenuous exercises, but otherwise they were allowed to follow their normal routine. The second group included 14 patients that had been admitted to a general internal medicine ward of a teaching hospital in Madrid (Spain). To be considered suitable for inclusion, patients were required to be over 18 and under 85 years of age, to have been admitted to a hospital for less than a week, and to have had at least a standard temperature recording above 38 ${}^{{}^{\circ}}$C the day before they were monitored. Temperature monitoring was carried out with two probes, placed in the external auditory canal (Mono-a-Therm Tympanic Temperature Probe, Mallinckrodt) for central temperature and in the cubital aspect of the forearm (Mono-a-Therm Skin Temperature Probe, Mallinckrodt) for peripheral temperature. Measurements were obtained once per minute during 24 h and stored in a holter device (TherCom©, Innovatec).

For the purposes of this work, an 8 h interval starting at 8:00 a.m. was selected. This way, the records were more uniform in terms of chonobiological effects, and this was an interval available in all the time series. Recordings from healthy individuals are labelled as Class 0 and recordings from patients are labelled as 1. These records have been used in previous publications by our group, where further details can be found [20]. Ethical Review Board approval was granted, and written informed consent gathered from each participant before inclusion.

## 3. Experiments and Results

The length of the time series was fixed at L=480, the 8 h interval stated above. ApEn and SampEn were also first tested using different values for their input parameters in the vicinity of the usual recommended configuration of r∈[0.1,0.2] and m=2 [47]. Specifically, the values for *m* were 1 and 2, and *r* varied between 0.1 and 0.25 in 0.05 steps. Except for r=0.1, with a relatively low classification performance of 64%, all the tested parameter values yielded a very similar accuracy, around 70%, with m=1 and r=0.25 offering a slightly superior performance. This final parameter configuration is very similar to that used in previous similar studies [5].

The influence of the embedded dimension on PE was also analysed, with *m* ranging from 3 up to 8. The classification results for each value are shown in Table 1. The value for the embedded dimension in PE was finally set at 8. This configuration was found to be optimal for the same time series in terms of classification performance and computational cost [48]. However, since m=8 does not satisfy the recommendation m!<<N, m=5 was also used in the computation of the final model.

The three statistics, ApEn (m=1,r=0.25,L=480), SampEn (m=1,r=0.25,L=480), and PE (m=8,L=480), were computed for each record first. Results are shown in Table 2.

The next step was to assess the independence between the input variables used to build the model. This step was carried out using a correlation matrix and by computing the *p*-values of the correlation test between variable pairs, as described in Table 3 and Table 4. This correlation analysis was used to assess the association degree between the information provided by ApEn and SampEn, in order to omit possible redundancy in the models fitted, and provide a rationale for not using both measures in the same model.

As expected, ApEn and SampEn are strongly correlated. However, PE exhibits very low correlations, and high *p*-values, which suggests there is no correlation between PE and any of the other two measures, ApEn or SampEn. This may be due to the fact that PE is based on ordinal differences, whereas the other two are based on amplitude differences, as was hypothesized.

In the following sections, the predictive capability of each one of the measures is assessed, using a logistic model for all the variables and their combinations, discarding the correlated cases. PE, ApEn, or SampEn are the temperature time series features used for classification.

### 3.1. Individual Models

Table 5 shows the results of the model using only PE. This model, whose assessment parameters are summarized in Table 6, achieves a significant classification performance, with 83.3% correctly classified records (Table 7) and an average classification performance of 77.6% using the LOO method (Table 6). The LOO method leaves out one time series (validation set) of each class, and a model is built using the remaining data (training data). This model is used to make a prediction about the validation set, and the final classification performance using LOO is obtained by averaging all the partial results. The classification achieved is expected to be lower than that for the entire dataset since training and test sets are different, but provides a good picture of the generalization capabilities of the model.

The percentages in Table 7 account for sensitivity (correct percentage for Class 0), specificity (correct percentage for Class 1), and classification accuracy (total). This will be repeated for the other models (confusion matrix).

Replacing the values obtained for the model coefficients, p(z) can then be computed as

$$p\left(z\right)=\frac{\exp\left(-32.202+3.704z_{\mathrm{PE}}\right)}{1+\exp\left(-32.202+3.704z_{\mathrm{PE}}\right)}$$.

For illustrative purposes, Table 8 shows the p(z) values obtained for all the PE results ($z_{\mathrm{PE}}$) in Table 2. If p(z)>0.5, time series should be classified as 1 or 0. According to this threshold, there are 2 classification errors in Class 0, and 3 in Class 1. This process can be repeated for all the models fitted in this study.

The results of the model using only SampEn are shown in Table 9. In contrast to results with PE, this model, whose parameters are summarized in Table 10, achieves a borderline classification performance instead, with 70% correctly classified records (Table 11), but only 57.1% for Class 1 records. The average classification performance was 68.7% using the LOO method (Table 10).

The last individual model, using only ApEn, achieves a better performance than that of SampEn. Its modelization results are shown in Table 12, and summary in Table 13. The classification performance is also at the verge of significance: 0.014 and 0.021, with an overall accuracy of 73.3%, but with better Class 1 classification, 64.3% (Table 14). The average classification performance was 69.7% using the LOO method (Table 10). The better performance of ApEn over SampEn using temperature records, although counter–intuitive, is in accordance with other similar studies [5].

### 3.2. Joint Models

The joint models correspond to models where PE and SampEn, or PE and ApEn, are combined to improve the classification performance of the models described in the previous section. The model results using PE and SampEn are shown in Table 15, Table 16 and Table 17. In comparison with previous individual results for PE or SampEn, there is a compelling performance improvement, from 83.3% to 90% classification accuracy, although the performance for SampEn was only 70%. Arguably, there is a synergy between PE and SampEn, as expected. The average classification performance was 87.2% using the LOO method (Table 16).

**Figure 1:**
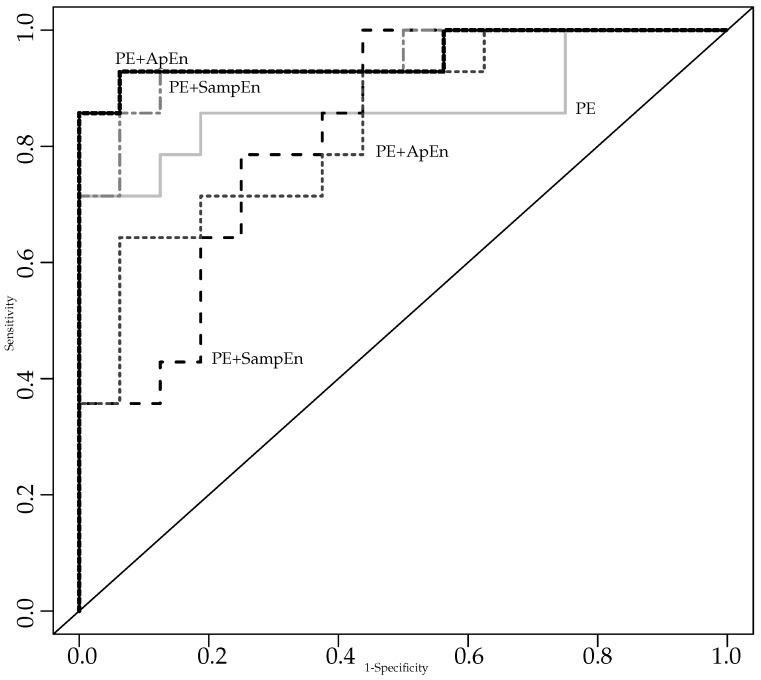
ROC plots of all the models studied. The best classification performance is achieved with the PE+ApEn model (bold dotted line).

Figure 1 summarizes the ROC plots of all the models studied. It becomes apparent in this figure how the performance significantly increases for the joint models.

**Figure 2:**
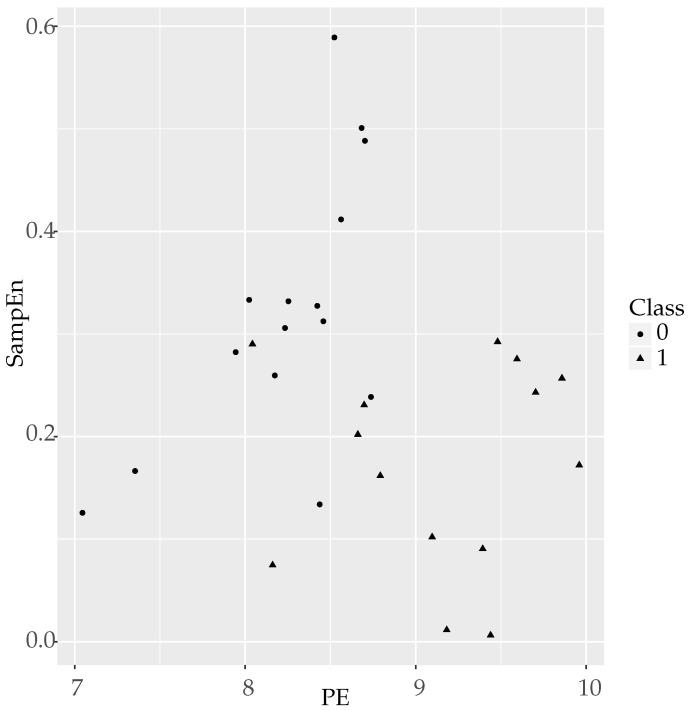
Clouds of points for temperature records using PE and SampEn as coordinates. The separability of the two classes can be easily observed, with Class 1 objects (triangles) located mainly at the lower right zone of the plot, whereas Class 0 objects (circles) are located at the higher left zone. Only two circles and one triangle are clearly misplaced, accounting for the errors in Table 17.

Visually, the separability of the classes using SampEn and PE combined in a logistic model, is shown in Figure 2. As numerically described in Table 17, only 1 or 2 objects are located in the opposite group.

The model results using PE and ApEn are shown in Table 18, Table 19 and Table 20. As for PE with SampEn, in comparison with previous individual results, there is a compelling performance improvement, from 83.3% to 93.3% classification accuracy, although the performance for ApEn was 73.3%. Again, there appears to be a synergy between PE and ApEn. The average classification performance was 90.1% using the LOO method (Table 19).

This is the model with the highest classification accuracy. Replacing the values obtained, the fitted logistic model becomes

(9) $$p\left(z\right)=\frac{\exp\left(-24.94++3.433z_{\mathrm{PE}}-12.806z_{\mathrm{ApEn}}\right)}{1+\exp\left(-24.94++3.433z_{\mathrm{PE}}-12.806z_{\mathrm{ApEn}}\right)}$$

from which p(z) can be computed replacing $z_{\mathrm{PE}}$ and $z_{\mathrm{ApEn}}$ by their results for each time series, as done for the univariate PE model.

**Figure 3:**
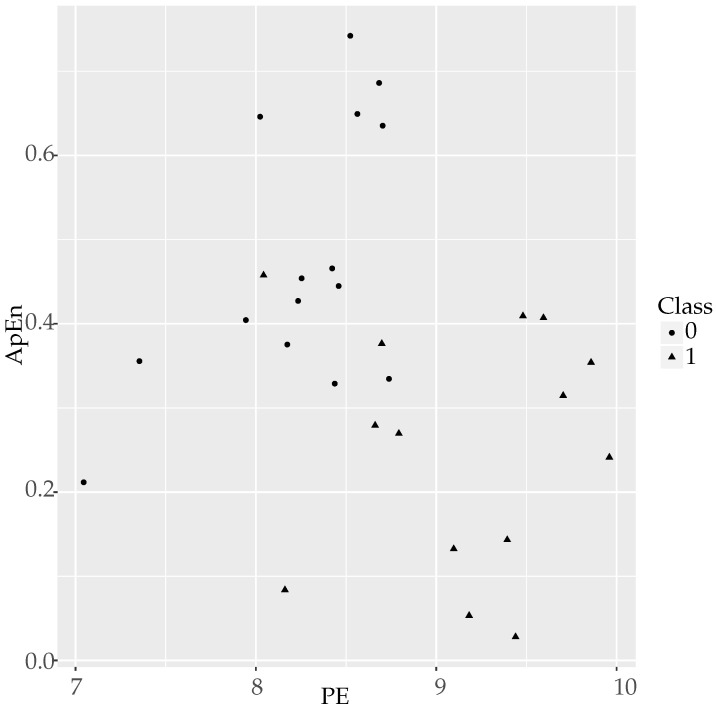
Clouds of points for temperature records using PE and ApEn as coordinates. The separability of the two classes can be easily observed, with Class 1 objects (triangles) located mainly at the lower right zone of the plot, whereas Class 0 objects (circles) are located at the higher left zone, as for the PE-SampEn case. Only one circle and one triangle are clearly misplaced, accounting for the errors in Table 20.

The separability of the classes using ApEn and PE combined in a logistic model is depicted in Figure 3. As numerically described in Table 20, only one object of each class is located in the opposite group.

The LOO analysis was also performed using these joint models, omitting a record from each class in each experiment, and averaging the classification results obtained. For the case with PE and ApEn, classification accuracy dropped from 90% to 87.22%. For the second model, it also dropped, from 93.3% to 90.11%. These performance decrements can be expected in any LOO analysis. A 3% difference can be considered small enough to assume a reasonable generalization capability for the joint models. Table 21 summarizes the performance of all the models studied.

The computation of the final model was repeated using the PE results achieved using m=5, as described in Table 1. In this case, the parameters of the model became $b_{1_{\mathrm{PE}}}=3.1462,$ $b_{2_{\mathrm{ApEn}}}=-14.54,b_{0}=-16.005$, with p=0.0000. Using this model instead, there were 4 classification errors in Class 0, and 1 error in Class 1, with a global accuracy of 83.3%. This is the same performance using only PE, but with a more conservative approach in terms of *m*. The classification was also improved by 10% in comparison with the results achieved by PE and ApEn in isolation with the same parameter configuration.

Finally, in order to further validate the approach proposed in this study, we applied the same scheme to EEG records of the Bonn database [49]. This database is publicly available and has been used in many studies, including ours [7,48,50] and others that have also proposed using more than an entropy statistic simultaneously [16,17] to improve classification performance. Therefore, we omit the details of this database, since it is not the focus of the present study, which can be obtained from those papers.

We applied the same SampEn and ApEn configuration as in [17], and the PE configuration used here. There is a great classification performance variation for each pair of classes, but the segmentation of EEGs from healthy subjects with eyes open (Group A in [17]) and from subjects with epilepsy during a seizure-free period from the epileptogenic zone (Group C in [17]) yielded a borderline significant classification performance (52% for PE, 78% for SampEn, and 72% for ApEn) that suited very well the case studied in the present paper.

A model including PE and SampEn was created as described above, with the following results: $b_{1_{\mathrm{PE}}}=-5.4482,b_{2_{\mathrm{SampEn}}}=39.2787,b_{0}=13.2617$, with p<0.01. Applying that model in a similar way as that in Equation (9), there were only 7 objects of Group A and 4 of Group C that were misclassified. Overall, the classification performance was increased up to 94.5%.

## 4. Discussion

PE could be initially supposed to look at signal properties that are different from those that ApEn or SampEn do. Indeed, the correlation analysis in Table 3 present very low values (–0.2374 and –0.1342), whereas, as expected, ApEn and SampEn were strongly correlated. This initial test suggested that only models with PE and either SampEn or ApEn should be studied. In addition, all the coefficients obtained were reasonably similar, without very large standard errors, which confirms that the S-shaped logistic model function is a suitable relationship for the data (there are no separation problems [51]).

Individual models were first computed for each measure in order to assess their performance independently. The classification results are acceptable for PE, but not for ApEn or SampEn, at most, borderline. PE results were 87.5% and 78.6%, but only 81.3% and 64.3% for ApEn and 81.3% and 57.1% for SampEn. While the classification accuracy for Class 0 is similar in all measures, it is very poor for Class 1 using ApEn or SampEn.

Two joint models were studied using PE and ApEn, and PE and SampEn, namely, by using pairs of the uncorrelated explanatory variables according to Table 3. The model with PE and ApEn improved the best individual performances in all cases, up to 93.8% and 92.9%. The model with PE and SampEn also improved the individual results, but to a lesser extent: 87.5% and 92.9%. Therefore, the classification results indicate that PE and ApEn are the best choice for a model in this case, confirmed by the minimum value achieved by the AIC (Table 21). According to these results, ApEn outperforms SampEn, which may seem counter-intuitive, but this also happened in a similar study with temperature records [5]. Moreover, the LOO analysis yielded a very similar classification performance, with only a 3% drop and still well above the individual performances. Specifically, the classification dropped from 83.3% to 77.6% using PE, from 70% to 68.7% using SampEn, and from 73% to 69.7% using ApEn. Regarding joint models, it also dropped from 93.3% to 87.2% using PE and ApEn, but with PE and SampEn it was fairly constant: 90% against 90.1%. Therefore, it can be concluded that the models are able to generalize well, given the small dataset available.

The Nagelkerke $R^{2}$ coefficients were smaller than 0.5 for the individual models using ApEn or SampEn only (0.493 and 0.399 respectively), whereas for PE, it was 0.588. These values also confirm that the individual results can only be considered significant for PE, although ApEn almost reached a level of significance in terms of $R^{2}$. The two joint models also improved with regard to this parameter, with values higher than 0.77.

In terms of class balance, the individual model based solely on SampEn yields 6 and 3 errors for each class—slightly unbalanced. However, results are more equally distributed for the other two individual models (3 and 2 errors for PE, and 5 and 3 errors for ApEn). For the joint models, there is a balanced classification, with 2 and 1 errors, or even 1 error for each class for the model proposed. This can be considered another advantage of the method proposed, since the classification is not only more accurate but also more equally distributed.

## 5. Conclusions

Entropy measures are sometimes unable to find significant differences among time series from disjoint clusters. This can be due to a sub-optimal parameter configuration, specific signal features, or simply because the method chosen is not appropriate for that purpose in that specific context. However, despite not finding statistically significant differences, classification results are frequently well over simple guessing, and such results are almost meaningful. Taking advantage of the fact that each measure is usually more focused on a specific region of the parameter space, we hypothesized that a combination of uncorrelated statistics could arguably improve the individual classification results achieved by each one independently and reach a suitable significance level.

With that purpose in mind, we analyzed the classification performance of a logistic model built from two entropy statistics, PE and ApEn/SampEn. These two measures look at different relationships of the information in the time series: ordinal or amplitude variations. Separately, they were less capable of tackling the difficult problem of body temperature time series classification (83% and 73% accuracy, respectively), but together the accuracy of the classification rose to 93% and 90% using a LOO approach. It is important to note that the main goal of this work was not to determine the exact percentage of correctly assigned objects was not the main goal of this work but to demonstrate that a combined approach can improve the baseline performance, however high or low it is already.

This scheme could be applied to other classification problems where independent measures achieve borderline results if applied in isolation. The exploitation of possible synergies between different methods is a novel approach that has not been applied very extensively so far, and could open doors to more accurate methods.

## Figures and Tables

**Table 1:** Classification results using permutation entropy (PE) with *m* ranging from 3 up to 8.

*m*	Sensitivity (Class 0)	Specificity (Class 1)	Accuracy (Correct)
8	0.875	0.786	0.833
7	0.9375	0.6428	0.8
6	0.75	0.7142	0.733
5	0.8125	0.5714	0.7
4	0.6875	0.5714	0.633
3	0.5625	0.6428	0.6

**Table 2:** Individual results for each of the measures employed.

	ApEn	PE	SampEn	Class
1	0.375369	8.173881	0.259592	0
2	0.355621	7.354433	0.166436	0
3	0.427184	8.233846	0.305818	0
4	0.328920	8.437123	0.133882	0
5	0.742144	8.523112	0.589070	0
6	0.444839	8.458743	0.312369	0
7	0.444839	8.253519	0.331899	0
8	0.465783	8.422755	0.327447	0
9	0.649292	8.562641	0.411671	0
10	0.334577	8.737973	0.238648	0
11	0.404367	7.944453	0.282284	0
12	0.686112	8.683103	0.500797	0
13	0.211678	7.045437	0.125658	0
14	0.635363	8.702552	0.488307	0
15	0.375369	8.173881	0.259592	0
16	0.646044	8.023478	0.333306	0
17	0.132585	9.096728	0.102007	1
18	0.409274	9.480602	0.292390	1
19	0.083815	8.160992	0.074664	1
20	0.457642	8.042545	0.290163	1
21	0.143293	9.393343	0.090606	1
22	0.354052	9.858080	0.256855	1
23	0.407061	9.594236	0.275580	1
24	0.314689	9.703521	0.243019	1
25	0.269602	8.792517	0.161793	1
26	0.027989	9.439494	0.006332	1
27	0.376410	8.697850	0.230783	1
28	0.241392	9.959548	0.172143	1
29	0.053292	9.182114	0.011505	1
30	0.279302	8.660942	0.201991	1

**Table 3:** Correlation results obtained for the three measures. Clearly, approximate entropy (ApEn) and sample entropy (SampEn) are strongly correlated.

	ApEn	PE	SampEn
ApEn	1.0000	−0.2374	0.9604
PE	−0.2374	1.0000	−0.1342
SampEn	0.9604	−0.1342	1.0000

**Table 4:** Significance of the correlation analysis between measures.

	ApEn	PE	SampEn
ApEn	-	0.2066	<0.0001
PE	0.2066	-	0.4795
SampEn	<0.0001	0.4795	-

**Table 5:** Variables in the equation for PE.

	Coefficients	Standard Error	Wald	df	Significance	Exp(B)
PE ($b_{1}$)	3.704	1.395	7.050	1	0.008	40.598
$b_{0}$	−32.202	12.025	7.171	1	0.007	0.000

**Table 6:** Individual model summary for PE. It includes some $R^{2}$ measures to assess the model’s predictive power, the area under ROC curve (AUC), and the leave-one-out (LOO) average classification results.

Step	−2 Log Likelihood	Cox–Snell $R^{2}$	Nagelkerke $R^{2}$	AUC	LOO
1	24.031	0.441	0.588	0.87	77.6%

**Table 7:** Percentage agreement between observed and predicted classifications for temperature records using an individual model based on PE.

		Predicted		
		Class		Percentage
Observed		0	1	Correct
Class	0	14	2	87.5
	1	3	11	78.6
**Total**				**83.3**

**Table 8:** Quantitative model probability results. Classification errors based on the computed p(z) value are in bold.

	PE	Class	p(z)
1	8.173881	0	0.1270
2	7.354433	0	0.0069
3	8.233846	0	0.1537
4	8.437123	0	0.2783
5	8.523112	0	0.3465
6	8.458743	0	0.2946
7	8.253519	0	0.1634
8	8.422755	0	0.2677
9	8.562641	0	0.3803
**10**	**8.737973**	**0**	**0.5402**
11	7.944453	0	0.0585
12	8.683103	0	0.4895
13	7.045437	0	0.0022
**14**	**8.702552**	**0**	**0.5075**
15	8.173881	0	0.1270
16	8.023478	0	0.0769
17	9.096728	1	0.8161
18	9.480602	1	0.9484
**19**	**8.160992**	**1**	**0.1218**
**20**	**8.042545**	**1**	**0.0821**
21	9.393343	1	0.9301
22	9.858080	1	0.9867
23	9.594236	1	0.9655
24	9.703521	1	0.9767
25	8.792517	1	0.5898
26	9.439494	1	0.9404
27	8.697850	1	0.5032
28	9.959548	1	0.9909
29	9.182114	1	0.8589
**30**	**8.660942**	**1**	**0.4690**

**Table 9:** Variables in the equation for SampEn.

	Coefficients	Standard Error	Wald	df	Significance	Exp(B)
SampEn ($b_{1}$)	−12.463	5.057	6.075	1	0.014	0.000
$b_{0}$	2.861	1.276	5.026	1	0.025	17.481

**Table 10:** Individual model summary for SampEn. It includes some $R^{2}$ measures to assess the model’s predictive power, the area under ROC curve (AUC), and the leave-one-out (LOO) average classification results.

Step	−2 Log Likelihood	Cox–Snell $R^{2}$	Nagelkerke $R^{2}$	AUC	LOO
1	30.798	0.299	0.399	0.82	68.7%

**Table 11:** Percentage agreement between observed and predicted classifications for temperature records using an individual model based on SampEn.

		Predicted		
		Class		Percentage
Observed		0	1	Correct
Class	0	13	3	81.3
	1	6	8	57.1
**Total**				**70.0**

**Table 12:** Variables in the equation for ApEn.

	Coefficients	Standard Error	Wald	df	Significance	Exp(B)
ApEn ($b_{1}$)	−11.744	4.761	6.083	1	0.014	0.000
$b_{0}$	4.096	1.770	5.354	1	0.021	60.077

**Table 13:** Individual model summary for ApEn. It includes some $R^{2}$ measures to assess the model’s predictive power, the area under ROC curve (AUC), and the leave-one-out (LOO) average classification results.

Step	−2 Log Likelihood	Cox–Snell $R^{2}$	Nagelkerke $R^{2}$	AUC	LOO
1	27.636	0.369	0.493	0.83	69.7%

**Table 14:** Percentage agreement between observed and predicted classifications for temperature records using an individual model based on ApEn.

		Predicted		
		Class		Percentage
Observed		0	1	Correct
Class	0	13	3	81.3
	1	5	9	64.3
**Total**				**73.3**

**Table 15:** Results for the logistic regression model using SampEn and PE.

	Coefficients	Standard Error	Wald	df	Significance	Exp(B)
SampEn ($b_{2}$)	−15.814	7.621	4.306	1	0.038	0.000
PE ($b_{1}$)	3.564	1.532	5.409	1	0.020	35.293
$b_{0}$	−26.899	12.955	4.311	1	0.038	0.000

**Table 16:** Summary for the joint model using SampEn and PE. It includes some $R^{2}$ measures to assess the model’s predictive power, the area under ROC curve (AUC), and the leave-one-out (LOO) average classification results.

Step	−2 Log Likelihood	Cox–Snell $R^{2}$	Nagelkerke $R^{2}$	AUC	LOO
1	15.396	0.580	0.775	0.95	87.2%

**Table 17:** Percentage agreement between observed and predicted classifications for temperature records using the joint model with SampEn and PE.

		Predicted		
		Class		Percentage
Observed		0	1	Correct
Class	0	14	2	87.5
	1	1	13	92.9
**Total**				**90.0**

**Table 18:** Results for the logistic regression model using ApEn and PE.

	Coefficients	Standard Error	Wald	df	Significance	Exp(B)
ApEn ($b_{2}$)	−12.806	6.457	3.934	1	0.047	0.000
PE ($b_{1}$)	3.433	1.602	4.590	1	0.032	30.974
$b_{0}$	−24.940	13.711	3.309	1	0.069	0.000

**Table 19:** Summary for the joint model using ApEn and PE. It includes some $R^{2}$ measures to assess the model’s predictive power, the area under ROC curve (AUC), and the leave-one-out (LOO) average classification results.

Step	−2 Log Likelihood	Cox–Snell $R^{2}$	Nagelkerke $R^{2}$	AUC	LOO
1	14.813	0.589	0.786	0.94	90.1%

**Table 20:** Percentage agreement between observed and predicted classifications for temperature records using the joint model with ApEn and PE. Results from previous experiments are included for comparative purposes.

		Predicted		
		Class		Percentage
Observed		0	1	Correct
Class	0	15	1	93.8
	1	1	13	92.9
**Total**				**93.3**
				**90.0 (87.5, 92.9) (SampEn, PE)**
				**73.3 (81.3, 64.3) (ApEn)**
				**70.0 (81.3, 57.1) (SampEn)**

**Table 21:** Best model fit based on the Akaike information criterion (AIC) for each case.

Explanatory Variable	Residual Variance	AIC
PE	24.03126	28.03126
ApEn	27.63616	31.63616
SampEn	30.79841	34.79841
PE+ApEn	14.81316	20.81316
PE+SampEn	15.39607	21.39607
